# Global eligibility and cost effectiveness of icosapent ethyl in primary and secondary cardiovascular prevention

**DOI:** 10.3389/fcvm.2023.1220017

**Published:** 2023-08-31

**Authors:** Peter P. Toth, Jean Ferrières, Max Waters, Martin Bødtker Mortensen, Nick S. R. Lan, Nathan D. Wong

**Affiliations:** ^1^ CGH Medical Center, Sterling, IL, United States; ^2^ Cicarrone Center for the Prevention of Cardiovascular Disease, Johns Hopkins University School of Medicine, Baltimore, MD, United States; ^3^ Department of Cardiology, Toulouse Rangueil University Hospital, Toulouse University School of Medicine, Toulouse, France; ^4^ Department of Cardiology, University Hospital Limerick, Limerick, Ireland; ^5^ Departments of Clinical Medicine & Cardiology, Aarhus University Hospital, Aarhus, Denmark; ^6^ Department of Cardiology, Fiona Stanley Hospital, Murdoch, WA, Australia; ^7^ Medical School, The University of Western Australia, Crawley, WA, Australia; ^8^ Division of Cardiology, University of California, Irvine, CA, United States

**Keywords:** eicosapentaenoic acid, cardiovascular disease, cost effectiveness, icosapent ethyl, eligibility

## Abstract

Icosapent ethyl (IPE) is a purified eicosapentaenoic acid–only omega-3 fatty acid that significantly reduced cardiovascular (CV) events in patients receiving statins with established cardiovascular disease (CVD) and those with diabetes and additional risk factors in the pivotal REDUCE-IT trial. Since the publication of REDUCE-IT, there has been global interest in determining IPE eligibility in different patient populations, the proportion of patients who may benefit from IPE, and cost effectiveness of IPE in primary and secondary prevention settings. The aim of this review is to summarize information from eligibility and cost effectiveness studies of IPE to date. A total of sixteen studies were reviewed, involving 2,068,111 patients in the primary or secondary prevention settings worldwide. Up to forty-five percent of patients were eligible for IPE, depending on the selection criteria used (ie, REDUCE-IT criteria, US Food and Drug Administration label, Health Canada label, practice guidelines) and the population studied. Overall, eight cost-effectiveness studies across the United States, Canada, Germany, Israel, and Australia were included in this review and findings indicated that IPE is particularly cost effective in patients with established CVD.

## 1. Introduction

Cardiovascular disease (CVD) is the most common cause of morbidity and mortality worldwide (1). Low-density lipoprotein cholesterol (LDL-C) continues to be the main treatment target for managing CVD risk (2), and statin therapy remains the cornerstone of preventive treatment for CVD. However, despite the effectiveness of statins, many patients receiving statin therapy continue to have a high residual cardiovascular (CV) risk (3). Consequently, research efforts are increasingly focused on discovering other therapeutic targets to help lower this persistent risk (4).

Mounting evidence from Mendelian and observational studies indicate that elevated triglyceride (TG) levels signal a substantial persistent residual risk for CVD (5). Nevertheless, until recently, no large, multinational, randomized trial in patients with elevated TG levels, including trials of niacin, fibrates, and mixed-omega-3 fatty acids comprising a combination of eicosapentaenoic acid (EPA) and docosahexaenoic acid, consistently showed improvement in CV outcomes in combination with statin therapy (6–9). This changed with the release of the pivotal REDUCE-IT study, a placebo-controlled, randomized, CV outcomes trial of the highly purified omega-3 stable ethyl ester of EPA, icosapent ethyl (IPE) in primary and secondary prevention settings (10, 11).

### 1.1. Eicosapentaenoic acid and cardiovascular disease outcomes

The REDUCE-IT trial included patients receiving statin therapy with fasting TG levels of 150 (with allowance for TG levels at or below 135 mg/dl due to intraindividual variability) to 499 mg/dl and LDL-C levels of 41 to 100 mg/dl who were aged forty-five years or older with established CVD or aged fifty years or older with diabetes mellitus and at least one additional risk factor (10). Select exclusion criteria were heart failure or renal impairment that was severe, severe active liver disease, glycated hemoglobin level higher than 10%, planned coronary surgery or intervention, history of chronic/acute pancreatitis, known hypersensitivity to shellfish or fish, or hypersensitivity to ingredients of icosapent ethyl or the placebo. The primary efficacy endpoint included a composite of CV death, nonfatal stroke, nonfatal myocardial infarction (MI), unstable angina, or coronary revascularization; the secondary endpoint was a composite of CV death, nonfatal stroke, or nonfatal MI (10).

After a median follow-up of 4.9 years, the primary efficacy endpoint occurred in 17.2% of patients in the IPE group vs. 22.0% of patients in the mineral oil placebo group (HR 0.75; *P *< 0.001), representing a twenty-five percent reduction in CV events with IPE. The key secondary endpoint occurred in 11.2% of patients receiving IPE vs. 14.8% receiving placebo (HR 0.74; *P *< 0.001) (10). With regard to safety, overall rates of adverse events were similar between IPE and placebo, albeit the rate of serious adverse bleeding events was nonsignificantly higher with IPE vs. placebo (2.7% vs. 2.1%, respectively; *P *= 0.06), and the rate of atrial fibrillation was significantly higher with IPE than with placebo (5.3% vs. 3.9%, respectively; *P *= 0.003). The US Food and Drug Administration (FDA) deemed that the benefits of IPE outweighed the potential risks. In secondary analysis, reductions in the primary and key secondary endpoints of REDUCE-IT were consistent across different statin background groups (12), suggesting that the type of statin used does not relevantly affect the efficacy of IPE.

Although IPE was first approved by the US FDA for lowering TG levels in patients with severe hypertriglyceridemia (11), the significant reduction in CV events reported in REDUCE-IT is unlikely to be attributed solely to lipid lowering because only a modest reduction in TG levels was observed (10). As such, other mechanisms of action of IPE are purported to be responsible for the significant reduction in CV events (10), including its effects on endothelial function, oxidative stress, foam-cell formation, inflammation/cytokines, plaque formation/progression, platelet aggregation, thrombus formation, and plaque rupture (13).

Since publication of the REDUCE-IT results, there has been much debate as to whether positive outcomes in REDUCE-IT can be attributed to the potential negative effects of mineral oil on lipid and inflammatory parameters in the placebo group (14, 15), namely increases in LDL-C (10.2%) and high-sensitivity C-reactive protein (29.9%; both *P *< 0.001). This controversy was particularly driven by a report that used data from the Copenhagen General Population Study to mimic lipid and high-sensitivity C-reactive protein trends of REDUCE-IT that found increased TG, LDL-C, and high-sensitivity C-reactive protein levels may be associated with increased risk of CVD in patients treated with mineral oil placebo (16). The assertion that changes in lipid levels and C-reactive protein were important contributors to the findings of REDUCE-IT has been independently investigated by multiple global regulatory authorities, including those in the United States and the European Union, both of which concluded that it is unlikely that mineral oil impacted CV outcomes in REDUCE-IT (17, 18), further adding that, even if it did have any effect, the effect was negligible, with EPA still providing at least a twenty-two percent incremental benefit over statin therapy alone (17). In addition, the JELIS trial, which assessed the efficacy of purified EPA 1.8 mg daily among 18,645 Japanese patients with hypercholesterolemia, did not use a mineral oil placebo, and yet reported similar reduction (nineteen percent) in CV events (19). Furthermore, mineral oil is a common agent used as a placebo in clinical trials due in part to its inert properties (20), and recent animal and *in vitro* data did not show significant changes in statin absorption when administered with mineral oil, nor any biologic properties of mineral oil (21, 22).

The positive CV outcomes in REDUCE-IT are further supported by findings in other trials that used EPA-only formulations (23), including JELIS (19), CHERRY (24), EVAPORATE (25), a Japanese study that reported reductions in CVD events after early initiation of EPA after percutaneous coronary intervention (PCI) in patients with acute coronary syndrome (26), and RESPECT-EPA (27–29).

Results from RESPECT-EPA were presented at the American Heart Association meeting in 2022 and the magnitude of the effect of EPA on its CV endpoints was consistent with the results of REDUCE-IT (27). The open-label trial included 2,460 Japanese patients treated with statins who were aged twenty to seventy-nine years with chronic coronary artery disease (CAD) and a low EPA-to-arachidonic acid ratio (<0.4); they were randomized in a one-to-one ratio to receive purified EPA 1.8 g/day plus statin therapy (*n* = 1,225) or statin monotherapy (*n* = 1,235). Purified EPA was associated with a borderline significant reduction of 21.5% in CV risk in the primary endpoint (*P *= 0.054) and a significant reduction of 26.6% in the secondary composite endpoint (*P *= 0.03) vs. statin monotherapy. Levels of EPA significantly increased from 48.5 at baseline to 140.5 µg/dl at the three-year follow-up in the EPA group vs. 46.6 to 51.5 µg/dl, respectively, in the statin monotherapy group (*P *< 0.05). Gastrointestinal disorders and new-onset atrial fibrillation occurred significantly (*P *< 0.001 and *P *= 0.017, respectively) more frequently in the EPA group than the statin monotherapy group (27). It is important to note here that CV clinical trials in Japan are open-label because blinding therapy is considered unethical (30).

### 1.2. Regulatory approval of icosapent ethyl for cardiovascular risk reduction and updates to guidelines

In the United States, the results from REDUCE-IT led the US FDA to grant IPE a second indication as an adjunct to maximally tolerated statin therapy to reduce the risk of MI, stroke, coronary revascularization, and unstable angina requiring hospitalization in adults with elevated TG levels (≥150 mg/dl) and either established CVD or diabetes mellitus with at least two additional risk factors for CVD (11). Soon after this approval, regulatory authorities in the European Union, United Kingdom, Canada, Hong Kong, Australia, and certain regions in the Middle East also approved IPE for CVD risk reduction (31, 32). In light of the data from REDUCE-IT, international medical societies and professional associations published scientific statements and updated guidelines, recommending use of IPE for CVD risk reduction (31, 33–38). These recommendations highlighted a need for IPE to be available worldwide.

Since the approval of IPE for residual CVD risk reduction, global interest has been growing to investigate the proportion of patients who may be eligible for and benefit from treatment with IPE in a real-world setting as well as its cost effectiveness in primary and secondary prevention settings (39–42). The aim of this review is to provide a comprehensive, worldwide overview of published, real-world eligibility analyses. A secondary aim is to provide an overview of published global cost-effectiveness data.

## 2. Eligibility for icosapent ethyl risk reduction in registry studies

### 2.1. Literature search

After the publication of REDUCE-IT in January 2019, PubMed and Google Scholar were continuously monitored for publications relating to eligibility for or cost effectiveness of IPE.

#### 2.1.1. Global and multiregional studies

##### 2.1.1.1. REACH registry

One of the larger studies on IPE eligibility was conducted using the REACH registry, which involved more than 65,000 patients closely resembling the patient population in REDUCE-IT (10); patients were aged forty-five years or older with stable atherothrombosis (ie, CAD, peripheral arterial disease, cerebrovascular disease) or with at least three risk factors for atherothrombosis and from forty-four countries across Asia, Australia, Europe, North and Central Americas, and the Middle East (Table 1) (41–60). Select REDUCE-IT inclusion criteria were applied to all patients with diabetes or atherosclerotic cardiovascular disease (ASCVD) and available baseline TG and total cholesterol levels. Overall, 11.3% (*n* = 7,085) patients were eligible for IPE, of whom 12.3% (*n* = 1,036) were eligible for IPE for primary prevention (41). The most common reasons for not meeting REDUCE-IT criteria included LDL-C levels above 100 mg/dl (60.8%), TG levels below 135 mg/dl (58.2%), and not receiving statin treatment (34.5%). After applying secondary prevention REDUCE-IT inclusion criteria to 54,046 with established ASCVD, 11.2% (*n* = 6,049) of patients were eligible for IPE (41). As in the primary prevention group, most patients who did not meet REDUCE-IT criteria had LDL-C levels above 100 mg/dl (53.5%), TG levels below 135 mg/dl (70.2%), and were not receiving statin treatment (35.3%). Patients eligible for IPE were at increased risk for cardiac atherothrombotic events vs. noneligible patients, including PCI and coronary artery bypass graft (CABG) surgery, demonstrating that persistent risk exists despite statin therapy (41).

**Table 1:** Eligibility for IPE risk reduction from cohorts by region.

Country	Data source	Patient population	Eligibility criteria	*N*	Eligibility for IPE
Global/multiregional					
45 countries across North, Central, and South Americas, Europe, Middle East, South Africa, Asia, and Australia	CLARIFY registry (43, 44)	Stable CAD	REDUCE-IT: Statin treatment; age ≥45 year; TG, ≥135 mg/dl and <500 mg/dl; LDL-C, >40 mg/dl and ≤100 mg/dl; established CVD or age ≥50 year with diabetes and ≥1 risk factor	24,146	15.5% eligible based on REDUCE-IT criteria
44 countries across North and Central Americas, Europe, Middle East, Asia, and Australia	REACH registry (41)	Age ≥45 year with established ASCVD or ≥3 ASCVD risk factors	REDUCE-IT: Statin treatment; age ≥45 year; TG, ≥135 mg/dl and <500 mg/dl; LDL-C, >40 mg/dl and ≤100 mg/dl; established CVD or age ≥50 year with diabetes and ≥1 risk factor	62,464	12.3% with diabetes mellitus eligible based on REDUCE-IT criteria 11.2% with established ASCVD eligible based on REDUCE-IT criteria
United States European Union	MESA CARDIA Dallas Heart Heinz Nixdorf Recall (45)	Hypertriglyceridemia without ASCVD	FDA IPE label: Statin treatment; diabetes and ≥2 risk factors	2,345	17% eligible based on FDA label for IPE
34 countries across North, South, and Central Americas, Europe, Africa, Asia, and Australia/New Zealand	VERTIS-CV (55, 56)	Diabetes mellitus and ASCVD	REDUCE-IT: Statin treatment; fasting TG, 135–499 mg/dl; LDL-C, 41–100 mg/dl	8,246	29.6% eligible based on REDUCE-IT criteria
34 countries across North, South, and Central Americas, Europe, Africa, Asia, and Australia/New Zealand	EMPA-REG OUTCOME (57, 58)	Type 2 diabetes and CVD	US FDA IPE label: TG, ≥150 mg/dl REDUCE-IT: Statin treatment; TG, 135–499 mg/dl; LDL-C, 41–100 mg/dl	7,020	45.3% eligible based on US FDA label for IPE 25.8% eligible based on REDUCE-IT criteria
North America					
United States	NHANES surveys (47)	Nonhospitalized and age >20 year	REDUCE-IT: Statin treatment; TG, 135–499 mg/dl; LDL-C, between 40 and 99 mg/dl; glycated hemoglobin, <10%; BP <200/100 mm Hg; established CVD or age ≥50 year with diabetes mellitus and ≥1 risk factor	21,548	2.8% eligible per REDUCE-IT criteria
United States	NHANES surveys (59)	Age ≥40 year who participated in laboratory component of survey	National Lipid Association (2019): Moderate to high intensity statin therapy; age ≥45 year; TG, 135–499 mg/dl; ASCVD or individuals age ≥50 year with diabetes and ≥1 additional CV risk factor, including hypertension, current cigarette smoking, low HDL-C, elevated high-sensitivity C-reactive protein, advanced age, kidney dysfunction, or presence of micro-/macro-albuminuria	2,729	7.8%^a^ or 6.8%^b^ per 2019 National Lipid Association
United States	VA health care cohorts (42)	ASCVD and age ≥45 year Diabetes mellitus without ASCVD and age >50 year	REDUCE-IT: TG, 150–499 mg/dl, LDL-C, 40–100 mg/dl, and ≥1 ASCVD risk factor for patients in the diabetes cohort Exclusion criteria: History of systolic heart failure, active liver disease/hepatic dysfunction, glycated hemoglobin >10%	1,695,750	14.5% with established ASCVD eligible based on REDUCE-IT criteria 17.1% with diabetes mellitus eligible based on REDUCE-IT criteria
Canada	Québec Heart Database (48)	History of CABG surgery	REDUCE-IT: Statin treatment; age ≥45 year; TG, 135–500 mg/dl; LDL-C, 41–100 mg/dl Health Canada IPE label: Statin treatment; TG, ≥135 mg/dl FDA IPE label: Statin treatment; TG, ≥150 mg/dl	12,641	21.9% eligible based on REDUCE-IT criteria 33.6% eligible based on Health Canada label 26.4% eligible based on FDA label
Canada	CANHEART cohort (49)	Established ASCVD	REDUCE-IT: TG, 135–499 mg/dl; LDL-C, 41–100 mg/dl	196,717	25.4% eligible based on REDUCE-IT criteria
Canada	Cohort of South Asian patients with ASCVD (50)	Established ASCVD	REDUCE-IT: Statin treatment; TG, >153 mg/dl and <500 mg/dl LDL-C, 40–100 mg/dl; established CVD or diabetes and ≥1 CV risk factor Health Canada IPE label: Statin treatment; elevated TG; established CVD or diabetes and ≥1 CV risk factor FDA IPE label: Statin treatment; TG, >150 mg/dl; established CVD or diabetes and ≥2 CV risk factors	200	33% eligible based on Health Canada and 2021 Canadian Cardiovascular Society dyslipidemia guidelines 25% eligible based on US FDA IPE label 17% eligible per REDUCE-IT criteria
European Union					
France	FAST-MI registry (51)	Acute MI	REDUCE-IT: Statin treatment; age ≥45 year; TG, 150–500 mg/dl; LDL-C, 40–100 mg/dl	9,459	12.5% based on REDUCE-IT criteria
Ireland	Single-center cohort (52)	Completion of cardiac rehabilitation 2018–2019 at a single center	REDUCE-IT: Statin treatment; age ≥45 year with established ASCVD or age ≥50 year with diabetes and ≥1 risk factor; TG, 135–499 mg/dl per initial protocol or 20–499 mg/dl per amended protocol; LDL-C, 41–100 mg/dl 2019 ESC/EAS guidelines: TG, 132.9–496.00 mg/dl	398	15.3% based on initial REDUCE-IT protocol 7.3% based on amended REDUCE-IT protocol 23.3% based on ESC/EAS guideline selection criteria
Denmark	Western Denmark Heart Registry (53)	Nonemergent symptoms suggestive of CAD undergoing CTA	REDUCE-IT: With or without diabetes; TG, 134.63–587.20 mg/dl; LDL-C, ≥41 to ≤100 mg/dl	23,759	9% eligible based on biochemical REDUCE-IT criteria
Pacific/Oceania					
Australia	Single-center cohort (54)	History of CABG surgery	REDUCE-IT: Statin treatment; age ≥45 year; TG, 150–499 mg/dl; LDL-C, 41–100 mg/dl	484	25.6% eligible based on REDUCE-IT criteria
Australia	Single-center cohort (60)	Diabetes and ACS	REDUCE-IT: Statin treatment; age ≥45 year; TG, 150–499 mg/dl; LDL-C, 41–100 mg/dl Exclusion criteria: Use of non-statin/ezetimibe medications; glycated hemoglobin >86 mmol/mol; pregnancy or breastfeeding; undergoing dialysis; severe liver disease; or systolic BP ≥200 mm Hg or diastolic BP ≥100 m Hg	205	22.9% per REDUCE-IT criteria 17.1% after applying REDUCE-IT exclusion criteria

ACS, acute coronary syndrome; ASCVD, atherosclerotic cardiovascular disease; BP, blood pressure; CABG, coronary artery bypass graft; CAD, coronary artery disease; CANHEART, Cardiovascular Health in Ambulatory Care Research Team; CARDIA, Coronary Artery Risk Development in Young Adults; CLARIFY, Prospective Observational Longitudinal Registry of Patients With Stable Coronary Artery Disease; CTA, computed tomography angiography; CV, cardiovascular; CVD, cardiovascular disease; EMPA-REG OUTCOME, Empagliflozin Cardiovascular Outcome Event Trial in Type 2 Diabetes Mellitus Patients; ESC/EAS, European Society of Cardiology/European Atherosclerosis Society; FAST-MI, French Registry of Acute ST Elevation or Non-ST-Elevation Myocardial Infarction; FDA, US Food and Drug Administration; IPE, icosapent ethyl; LDL-C, low-density lipoprotein cholesterol; MESA, Multiethnic Study of Atherosclerosis; MI, myocardial infarction; NHANES, National Health and Nutrition Examination Survey; REACH, Reduction of Atherothrombosis for Continued Health; REDUCE-IT, Reduction Of Cardiovascular Events with Icosapent Ethyl–Intervention Trial; TG, Triglycerides; VA, Veterans Affairs; VERTIS-CV, Evaluation of Ertugliflozin Efficacy and Safety Cardiovascular Outcomes Trial.

^a^ Assuming that statin use is contraindicated in those not on statins.

^b^ Assuming initiation and maximal escalation of lipid-lowering therapies.

##### 2.1.1.2. CLARIFY registry

The CLARIFY registry included 32,703 patients with stable CAD (eg, documented MI/CABG surgery/PCI >3 months ago) in forty-five countries across Africa, Asia, Australia, Europe, the Middle East, and North, Central, and South Americas (43, 44). After applying REDUCE-IT inclusion criteria to 24,146 patients with complete baseline data, 15.5% (3,738) of patients were eligible to receive IPE. Similar to those in the REACH registry (41), most patients who did not meet REDUCE-IT eligibility criteria had TG levels below 135 mg/dl (57.1%) and LDL-C levels above 100 mg/dl (34.4%); other ineligible patients had LDL-C levels at or below 40 mg/dl (12.6%), were younger than forty-five years of age (3.8%), or had TG levels at or above 500 mg/dl (0.6%). Extrapolating these results to the global (111 million) and US populations (17 million) with CAD, an estimated 17.14 million and 2.56 million patients with CAD, respectively, may be eligible for and benefit from treatment with IPE (43). It is important to note that the CLARIFY registry did not include US patients, many of whom are more likely to be eligible for IPE given the greater prevalence of high TG levels and use of intensive statin treatment. In addition, the REDUCE-IT trial included a broader patient population vs. the CLARIFY registry, which included only patients with CAD (41).

##### 2.1.1.3. VERTIS-CV

Kim et al. (56) used the global VERTIS-CV trial of 8,246 patients with diabetes mellitus and ASCVD to assess eligibility for IPE across four subgroups of patients stratified by baseline TG and LDL-C levels (TG < 135 mg/dl and LDL-C < 70 mg/dl; TG < 135 mg/dl and LDL-C ≥ 70 mg/dl; TG ≥135 mg/dl and LDL-C < 70 mg/dl; and TG ≥135 mg/dl and LDL-C ≥ 70 mg/dl). Mean patient age was sixty-four years, with median TG and LDL-C levels of 153 and 82 mg/dl, respectively. After applying the REDUCE-IT criteria, 29.6% (*n* = 2,444) of patients were eligible to receive IPE. Overall, 18.2% of patients were not receiving statins, and 65.3% had LDL-C levels of 70 mg/dl or higher, highlighting the gap in statin use and lipid management. In addition, 30.3% (*n* = 2,500) did not fully meet REDUCE-IT eligibility criteria, but they had TG levels above 135 mg/dl, and 3,302 (40.0%) did not meet REDUCE-IT eligibility criteria but had TG levels at or below 135 mg/dl. Adding IPE could have been considered in sixty percent of patients based on the REDUCE-IT trial inclusion criteria or on TG levels alone (56).

##### 2.1.1.4. MESA, CARDIA, the Dallas Heart, and the Heinz Nixdorf Recall studies

Using IPE eligibility per the US FDA label, Cainzos-Achirica (45) evaluated whether the coronary artery calcium (CAC) score could enhance risk stratification among individuals with hypertriglyceridemia in the primary prevention setting. The study pooled data from 2,345 patients from four studies, including MESA, CARDIA, the Dallas Heart, and the Heinz Nixdorf Recall studies, and evaluated the incidence of ASCVD events in patients meeting IPE eligibility criteria per the US package insert and stratified by CAC scores (0, >1–100, >100). The outcome of this study was designed to simulate that of the composite ASCVD endpoint in REDUCE-IT, which included CV death, nonfatal MI, unstable angina, coronary revascularization, or nonfatal stroke. Overall, seventeen percent (*n* = 643) of patients were eligible for IPE per the US FDA IPE label. After stratifying patients eligible for IPE by CAC category, twenty-five percent had a CAC score of zero with a five-year incidence of ASCVD of 7.2%; thirty-seven percent had a CAC score of one to 100 with a five-year incidence of ASCVD of 10.9%; and thirty-eight percent had a CAC score higher than 100 with a five-year incidence of ASCVD of 15.9%. The number of patients ineligible for IPE with a CAC score higher than one hundred (17%) was similar to that of participants eligible for IPE therapy irrespective of CAC score (17%), suggesting that trial enrollment and pharmacotherapy allocation approaches using the presence of diabetes, various additional risk factors, or both may miss a large portion of individuals with hypertriglyceridemia at high risk for ASCVD events who may benefit from IPE. Importantly, the study showed that long-term residual CV risk is substantial despite high-intensity statin treatment, suggesting that other therapies are needed to reduce this risk (45).

##### 2.1.1.5. EMPA-REG OUTCOME

Verma et al. (57) used data from the global EMPA-REG OUTCOME trial of empagliflozin in 7,020 patients with type 2 diabetes and established CVD. Eligibility for IPE was assessed using REDUCE-IT eligibility criteria and US package insert criteria. In addition, they investigated whether CV outcomes and efficacy of empagliflozin differed based on eligibility for IPE treatment. Overall, 1,810 (26%) patients (placebo, *n* = 608; empagliflozin, *n* = 1,202) fulfilled the REDUCE-IT criteria, and 3,182 (45%) patients (placebo, *n* = 1,043; empagliflozin, *n* = 2,139) fulfilled US FDA label requirements. Compared with those not fulfilling the criteria, these patients had generally comparable risk of CV outcomes, and the treatment effect of empagliflozin was comparable across all cohorts (57).

#### 2.1.2. United States

##### 2.1.2.1. NHANES 2017–2020

Using data from the National Health and Nutrition Examination Survey (NHANES) from 2017 to 2020, Shen et al. (59) investigated eligibility and use of lipid-lowering therapies, including statins, IPE, and proprotein convertase subtilisin/kexin type 9 inhibitors, among US adults. Eligibility criteria for IPE were based on the scientific statement from the National Lipid Association (33), which included patients treated with moderate- to high-intensity statins aged forty-five years or older with clinical ASCVD or aged fifty years or older with diabetes and at least one additional CV risk factor (eg, hypertension, current cigarette smoking, low high-density lipoprotein cholesterol, advanced age, residual TG 135–499 mg/dl even after statin treatment) (59). Of 2,729 sampled individuals (or 149.3 million US adults), forty-four percent (*n* = 1,376) had had an indication for statins (or 65.8 million US adults), but only forty-five percent of these had taken statins. Given that use of guideline-recommended lipid-lowering therapies such as statins is suboptimal, eligibility for IPE was assessed in two scenarios: (1) assuming existing lipid-lowering therapy as the maximum tolerated dose before evaluating eligibility and (2) assuming initiation and maximal escalation of preexisting lipid-lowering therapies and accounting for expected lipid improvements. Based on lipid profiles and existing therapies, 7.8% (11.6 million US adults) would benefit from IPE, including 29.2% (6.1 million US adults) of patients with ASCVD and 35.6% (8.9 million US adults) of patients with diabetes. Assuming maximal escalation of statins and addition of ezetimibe, 6.8% (10.2 million) of patients would benefit from IPE, including 26.0% (5.4 million) with ASCVD and 31.4% (7.8 million) with diabetes. Eligibility for IPE in this study was relatively higher than reported in other studies partly because the 2019 statement from the National Lipid Association did not include LDL-C criteria. Overall, the study underscored that use of lipid-lowering therapies is suboptimal (59).

##### 2.1.2.2. NHANES 1996–2016

Using the NHANES dataset from 1999 to 2016 involving 53,348 US adults, Wong et al. (47) applied IPE eligibility criteria per REDUCE-IT or the US package insert for IPE to estimate the proportion of US adults who may benefit from IPE and the number of potentially preventable ASCVD events with IPE therapy. The number of anticipated ASCVD events, including the REDUCE-IT primary composite endpoint, secondary composite endpoint, and individual secondary endpoints (CV-related death, revascularization, nonfatal MI, stroke, and total mortality), was estimated using the overall REDUCE-IT initial and total published event rates as well as event rates for initial events alone in the IPE and placebo groups. REDUCE-IT criteria were applied to 21,548 NHANES participants randomly assigned to morning sessions who fasted for at least 8.5 h. Overall, 319 participants (or projected 3,041,891 US adults) would be eligible to receive IPE, including 114 participants (*n* = 1,133,110) for primary prevention and 205 participants (*n* = 1,908,781) for secondary prevention. Ineligibility for IPE was most commonly due to age younger than forty-five years (*n* = 10,103) and TG levels below 135 mg/dl or 500 mg/dl or higher (*n* = 7,148). Treatment with IPE for more than 4.9 years could potentially prevent 71,391 primary and 31,660 secondary composite outcomes each year, with 29,798 and 22,349 accounting for first time events, respectively. The majority of estimated preventable events occurred in patients eligible for IPE in the secondary prevention setting vs. the primary prevention cohort (47).

After applying the US FDA IPE label inclusion criteria, 476 participants (or 4,564,056 US adults) were eligible to receive IPE, with 161 (*n* = 1,614,561) and 315 (*n* = 2,949,495) eligible in primary prevention and secondary prevention settings, respectively (47). The most common reasons for ineligibility included TG levels below 150 (*n* = 15,595), not receiving statins (*n* = 4,857), and not meeting criteria for primary prevention (*n* = 620). If these patients were given IPE for 4.9 years, then 60,544 and 41,915 primary and secondary composite endpoints, respectively, were estimated to be prevented each year (47).

##### 2.1.2.3. US Veterans Affairs healthcare system

Jia et al. (42) used primary (diabetes mellitus) and secondary prevention (prior MI, ischemic stroke, or peripheral artery disease) cohorts receiving care in the US Veterans Affairs Healthcare System between October 2013 and September 2014 to estimate the number of patients who may benefit from IPE per the REDUCE-IT eligibility criteria. Patients with available TG data, aged forty-five years or older with ASCVD (*n* = 1,011,558), or aged fifty years or older with diabetes mellitus (*n* = 684,192) were screened per REDUCE-IT inclusion and exclusion criteria. A total of 263,114 patients were eligible for IPE in the primary and secondary prevention settings: 17.1% (*n* = 116,925) of patients for primary prevention and 14.5% (*n* = 146,189) of patients for secondary prevention (42).

#### 2.1.3. Canada

##### 2.1.3.1. Québec Heart Database

Kosmopoulos et al. (48) used REDUCE-IT-derived, US FDA IPE package insert, and Health Canada IPE label selection criteria to determine IPE eligibility in the secondary prevention setting among 12,641 patients from the Québec Heart Database who underwent CABG surgery between 2006 and 2016. Overall, 21.9% (*n* = 2,769) of patients were eligible for IPE per the REDUCE-IT criteria. Reasons for ineligibility included TG levels below 135 or higher than 500 mg/dl (*n* = 7,217), LDL-C levels below 41 or higher than 100 mg/dl (*n* = 1,396), not treated with statins (*n* = 1,230), and age younger than forty-five years (*n* = 29). After applying Health Canada label selection criteria, which included patients receiving statins with TG levels 135 mg/dl or higher, 33.6% (*n* = 4,253) were eligible for IPE. Reasons for ineligibility included TG levels below 135 mg/dl (*n* = 7,158) and not receiving statins (*n* = 1,230). Per the US label selection criteria, 26.4% (*n* = 3,337) of patients were eligible for IPE; reasons for ineligibility included TG levels less than 150 mg/dl (*n* = 8,074) and not receiving statins (*n* = 1,230). Regardless of whether the REDUCE-IT–derived, Health Canada label, or US label selection criteria were applied, this study showed that a considerable proportion of patients would be eligible for and benefit from use of IPE as an adjunct to secondary prevention therapies after CABG surgery (48).

##### 2.1.3.2. CANHEART

Lawler et al. (49) used the CANHEART cohort of 9,403,853 patients across Ontario, Canada, to determine the real-world risk of ASCVD events in a secondary prevention setting involving patients with ASCVD and hypertriglyceridemia as well as to estimate the proportion of patients who may qualify for IPE therapy. Only patients with lipid panels, aged forty years or older, and a history of ASCVD but without major life-limiting conditions and those not living in a skilled nursing facility were screened for IPE eligibility per the REDUCE-IT criteria. Of 196,717 patients with established ASCVD, 25.4% (*n* = 49,886) were eligible for IPE. Over a median of three years, 24,097 composite ASCVD events occurred with a primary outcome (ie, unstable angina, first occurrence of MI, stroke, transient ischemic attack, coronary revascularization, CVD-related death) at a rate of 38.6 per 1,000 person-years. The event rate for patients with TG levels above 354.3 mg/dl was 52% higher than for patients with TG levels below 88.6 mg/dl, indicating that this patient population may benefit from therapies such as IPE that reduce residual CV risk (49).

##### 2.1.3.3. South Asian patients in community cardiology and family practice clinics

South Asians are particularly prone to CVD and CVD-related mortality (61–63), and, given that an overwhelming majority of patients in REDUCE-IT were White, Krishnaraj et al. (50) sought to determine the generalizability of the REDUCE-IT results in the secondary prevention setting—using the Health Canada IPE label, the Canadian Cardiovascular Society dyslipidemia guidelines, the US FDA IPE label, and REDUCE-IT criteria—to 200 South Asian patients treated with statins aged forty-five years or older with ASCVD from community cardiology and family practice clinics living in Canada. Overall, ninety-five percent of patients had CAD, eighty-six percent underwent coronary revascularization, forty percent had a history of MI, and seventy-five percent were taking high-dose statins. Per the Health Canada IPE label (64), IPE is indicated for patients with elevated TG levels, with increased CV risk due to established CVD, or diabetes and at least one other CV risk factor. Although the Health Canada label did not denote a TG threshold (64), Krishnaraj et al. (50) specified a TG threshold of 135 mg/dl or higher to represent hypertriglyceridemia. The Canadian Cardiovascular Society dyslipidemia guidelines recommend IPE for patients with TG levels 135 to 499 m/dl (37). Regardless of the slight differences in specified TG levels between the Health Canada IPE label and Canadian guidelines, patients eligible for IPE using either selection criteria was similar, with approximately thirty-three percent of patients eligible for IPE. After applying the US FDA IPE label TG threshold of 150 mg/dl or higher and the REDUCE-IT criteria for TG levels (135–499 mg/dl) and LDL-C levels (41–100 mg/dl), 24.5% and 17.0% of patients, respectively, would be eligible for IPE.

#### 2.1.4. France

##### 2.1.4.1. FAST-MI 2010 and 2015

Using the FAST-MI 2010 and FAST-MI 2015 registries comprising 9,459 patients with acute MI across France (65), Ferrières et al. (51) applied the REDUCE-IT selection criteria to 3,789 patients with a complete lipid panel; exclusion criteria were also applied. Overall, 12.5% (*n* = 472) of patients were eligible for IPE. Despite several differences between patients in the FAST-MI registries and REDUCE-IT trial, such as the prevalence of established ASCVD, cigarette smoking, hypertension, and statin use, their residual risk was similar with an event rate of 36.7 per 1,000 patient-years in the FAST-MI registry vs. 36.9 per 1,000 patient-years in REDUCE-IT. Overall, the proportion of patients eligible for IPE in this study was lower vs. other studies, in part because the FAST-MI registries only included patients hospitalized with acute MI rather than all patients with established CVD (51).

#### 2.1.5. Denmark

##### 2.1.5.1. Western Denmark Heart Registry

Using data from 23,759 patients aged eighteen years or older who presented with nonemergent symptoms suggestive of CAD and who underwent computed tomography angiography between January 2008 and December 2017 in the Western Denmark Heart Registry, Mortensen et al. (53) estimated ASCVD event rates (ie, MI, stroke, revascularization, all-cause mortality >90 days after testing), including hard ASCVD events (ASCVD events, excluding revascularization), and a five-year number need to treat (NNT) to prevent one ASCVD event in patients meeting IPE eligibility criteria, including patients with or without diabetes, TG levels 134.63 to 587.20 mg/dl, and LDL-C levels of at least 41 but no higher than 100 mg/dl. The ASCVD event rates were stratified by calcified plaque burden (CAC scores of 0, 1–299, and ≥300) and CAD severity defined by computed tomography angiography (no CAD, nonobstructive CAD, and obstructive CAD); patients with prior MI or revascularization were excluded. Overall, nine percent (*n* = 2,146) of patients were eligible for IPE: 523 patients with obstructive CAD, 738 patients with nonobstructive CAD, and 885 with no CAD. A considerable difference was observed in the estimated five-year NNT to prevent one event across increasing CAC scores among REDUCE-IT–eligible patients, ranging from eighty-seven in patients with a CAC score of zero to seventeen in patients with a CAC score above 300. In addition, the NNT for hard ASCVD events was lower in patients with extensive nonobstructive CAD vs. patients with obstructive CAD and lower plaque burden. Overall, the study found that REDUCE-IT-eligible patients with high plaque burden but nonobstructive CAD may benefit more from IPE than patients with obstructive CAD and less plaque burden (53).

#### 2.1.6. Ireland

##### 2.1.6.1. Single-center cardiac rehabilitation center

Using a single-center cardiac rehabilitation cohort population in Ireland, Gaine et al. (52) applied REDUCE-IT selection criteria (initial and amended) and the 2019 European Society of Cardiology/European Atherosclerosis Society guideline selection criteria to 275 patients with available LDL-C and TG data. The initial REDUCE-IT criteria for TG levels were 150 mg/dl or higher (with allowance for ≥135 mg/dl due to intraindividual variability), a value that was later amended to 200 mg/dl or higher (10). After applying the REDUCE-IT criteria, 15.3% (*n* = 42) and 7.3% (*n* = 20) of patients were eligible for IPE per the initial and amended REDUCE-IT criteria, respectively (52). According to the 2019 European Society of Cardiology/European Atherosclerosis Society guideline selection criteria for IPE (TG 132.86–496.00 mg/dl, no LDL-C or age limits), 23.3% (*n* = 64) of patients were eligible for IPE (52).

#### 2.1.7. Australia

##### 2.1.7.1. Tertiary hospital

Using data from 1,676 patients who underwent CABG surgery in a western Australian tertiary hospital from February 2015 until August 2020, Lan et al. (54) applied REDUCE-IT inclusion criteria to 484 patients with available follow-up lipid profiles. Patients not eligible for IPE were those not prescribed statin therapy or taking a fibrate. Only some REDUCE-IT exclusion criteria were applied due to limitations in data availability and retrospective analysis. In total, 25.6% (*n* = 124) of patients were eligible for IPE. Results from this study demonstrate that a substantial proportion of patients may benefit from therapy with IPE following CABG surgery (54).

##### 2.1.7.2. Tertiary hospital

Lan et al. (60) applied secondary prevention REDUCE-IT eligibility criteria in 205 patients with diabetes and acute coronary syndrome in a western Australian tertiary hospital. Several exclusion criteria were also applied, some of which included use of nonstatin or nonezetimibe medications, undergoing dialysis, severe liver disease or poorly controlled hypertension. Overall, 22.9% (*n* = 47) and 17.1% (*n* = 35) of patients were eligible for IPE based on REDUCE-IT inclusion only and after applying exclusion criteria, respectively. Importantly, the study found that nearly two-thirds of patients with diabetes who would be eligible for IPE had a prior history of ASCD, suggesting that targeting LDL-C alone for prevention is not sufficient (60).

## 3. Cost effectiveness of icosapent ethyl

Cost-effective, add-on therapies to statins are needed for primary and secondary CVD prevention to help reduce the incidence of CV events and the costs associated with these events. REDUCE-IT was the first and only trial to demonstrate a cardiovascular mortality benefit over and above a statin. However, providing IPE therapy to all eligible patients necessitates a substantial investment (66).

Although generic formulations of IPE are available and it is well established that generic drugs cost less than branded drugs, these formulations are not approved for the same indications as their branded counterparts. In the United States, generic IPE is approved for only one indication, ie, as an adjunct to diet to reduce TG levels in adults with severe (≥500 mg/dl) hypertriglyceridemia. Only branded IPE is approved for reducing TG levels and reducing CV events in primary and secondary prevention settings (11).

At the net annual price of $1,625 for IPE, approximately four percent of eligible patients could be treated in a year before exceeding the Institute for Clinical and Economic Review (ICER) budget impact threshold of $819 million (67). As, such, concerns regarding potential lack of access to IPE prompted the ICER to issue an access and affordability alert for IPE, signaling to stakeholders and policymakers “that the amount of added health care costs associated with a new service may be difficult for the health care system to absorb over the short term without displacing other needed services or contributing to rapid growth in health care insurance costs” that threaten sustainable access to high-value care for all patients (67). Thus, it is important to understand the value of IPE treatment in different populations so that health benefit from treatment can be maximized.

**Figure 1:**
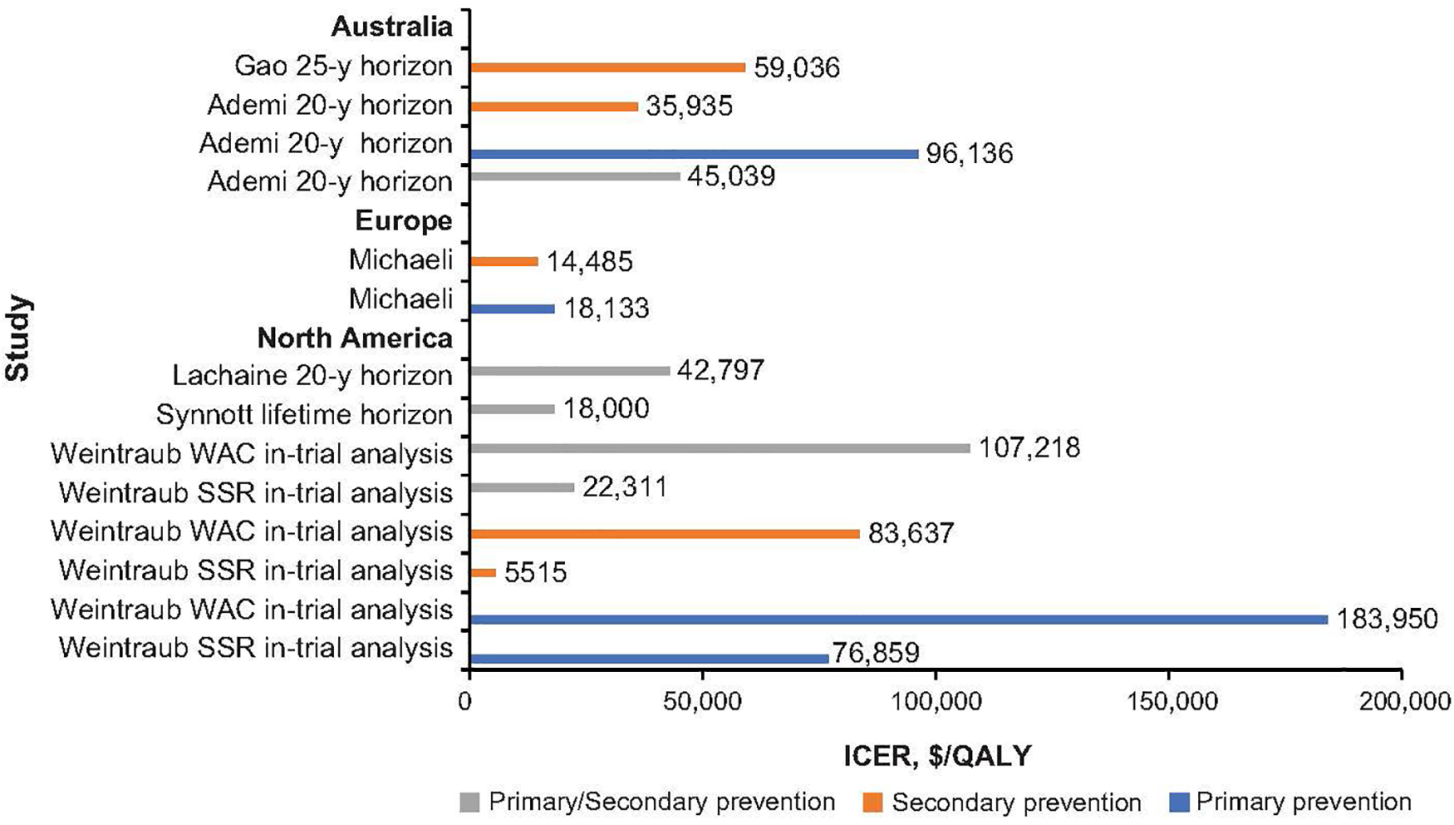
Incremental cost-effective ratio with icosapent ethyl across world regions. ICER, incremental cost-effectiveness ratio; QALY, quality-adjusted life-year; WAC, wholesale acquisition cost (40, 68–72).

Since the approval of IPE by various regulatory agencies, interest has been growing to understand the value of IPE as a treatment option, and multiple studies worldwide have investigated the cost effectiveness of IPE vs. standard of care in primary and secondary prevention settings (Table 2; Figure 1) (39, 40, 66, 68–72).

**Table 2:** Cost-effectiveness studies of IPE risk reduction by region.

Region	Analysis	Model	Time horizon	Annual price	Willingness-to-pay threshold	Results
North America						
United States (39)	CUA per QALY	Patient-level, in-trial cost, clinical outcomes, and long-term costs, events, and life expectancy derived from Markov simulation models	Lifetime	$1,518	$50,000 $100,000 $150,000	IPE costs less than in-trial SOC ($23,926 vs. 24,563) and over lifetime ($87,077 vs. 88,912); yielded more QALYs than SOC (3.34 vs. 3.27 in-trial and 11.61 vs. 11.35 lifetime) IPE was dominant strategy: in-trial (73.2%) vs. lifetime (71.6%) in simulations
United States (69)	CEA per QALY	In-trial CEA conducted using patient-level data from REDUCE-IT; lifetime CEA was conducted using Markov state-transition model, microsimulation, and data from REDUCE-IT and medical literature	In trial and lifetime	$1,518 (SSR Health) $3,387 (WAC)	$50,000	Within trial, ICER for IPE vs. SOC was $22,311/QALY gained using the SSR Health cost and $107,218/QALY gained using the WAC Over lifetime vs. SOC, IPE was projected to be cost saving using SSR Health cost but more costly using WAC Compared with SOC, IPE had a 58.4% probability of being less costly and more effective over lifetime using SSR Health cost, 89.4% probability of having an ICER <$50,000/QALY gained using SSR Health cost, and a 72.5% probability of having an ICER <$50,000/QALY gained using WAC
United States (68)	CUA and CEA (per QALY, LYG, and evLYG)	Markov model: patients began in treated state and could move to event states of MI, stroke, or death, and could then move into a postevent state	Lifetime	$1,625	$100,000	ICER: $18,000/QALY for IPE vs. medical management alone; $17,000/LYG and $17,000/evLYG
Canada (70)	CUA per QALY	Markov model with 5 health states: Event-free CV, nonfatal CV events (nonfatal MI, nonfatal stroke, coronary revascularization, or hospitalization for unstable angina), post-nonfatal CV event, death from fatal CV causes, and death from other causes	20 year	NA	$50,000	ICER: $42,797/QALY gained (SD 15,884)
Europe						
Germany (40)	CEA per QALY	Markov cohort model [3 health states: Alive without CVD, alive with CVD, and death (CVD death and non–CVD-related death)]	20 year	€2,400	€20,000	Primary CVD prevention: IPE + statin generated 0.81 additional QALYs at an incremental cost of €14,732, was cost effective (ICER: 18,133/QALY) Secondary CVD prevention: IPE + statin generated 0.99 additional QALYs at an incremental cost of €14,333, was cost effective (ICER: 14,485/QALY)
Middle East						
Israel (66)	NNT/CNT	NNT-/CNT-based analysis corresponding to annual budget impact threshold of ICER to estimate preventable MACE	NA	$2,915	NA	CNT to prevent 1 MACE: $842,726 for primary prevention $199,969 for secondary prevention IPE worth $819 million can avoid 20,069 MACEs for secondary prevention, 4,762 MACEs for primary prevention
Pacific/Oceania						
Australia (71)	CUA and CEA (per QALY/per YOLS)	Markov model with 3 health states: alive with CVD, alive without CVD, dead	20 year	AUD1,637	AUD50,000	ICER: AUD45,039 Primary prevention: AUD96,136/QALY AUD113,916/YOLS Secondary prevention: AUD35,935/QALY AUD29,250/YOLS
Australia (72)	CUA and CEA (per QALY/per LY)	Markov model with 4 health states: no further event, post-CVD, CVD death, and non–CVD-related death	25 year	AUD3,768	AUD50,000	ICUR: AUD59,036/QALY ICER: AUD54,358/LY

CEA, cost-effectiveness analysis; CUA, cost utility analysis; CNT, cost needed to treat; CV, cardiovascular; CVD, cardiovascular disease; evLYG, equal value of life-year gained; ICER; incremental cost-effectiveness ratio; ICUR, incremental cost-utility ratio; IPE, icosapent ethyl; LY, life-year; LYG, life-year gained; MACE, major adverse cardiovascular event; MI, myocardial infarction; NA, not available; NNT, number needed to treat; QALY, quality-adjusted life-year; REDUCE-IT, Reduction of Cardiovascular Events with Icosapent Ethyl–Intervention Trial; SOC, standard of care; WAC, wholesale acquisition cost; YOLS, year of life saved.

From US and Canadian health care perspectives, IPE is a cost-effective therapeutic option, falling at or below the willingness-to-pay threshold of $50,000 or 100,000, depending on the study (39, 68–70). Several studies indicate that IPE is cost effective for both primary and secondary prevention settings, but IPE consistently offers more value for secondary prevention (68, 70, 73). A US study by Weintraub et al. showed that probability of cost effectiveness of IPE over a lifetime, below a willingness-to-pay threshold of $50,000, was 89% and 72% depending on different cost used for IPE in the model (69).

Cost effectiveness of IPE from a German health care perspective was consistent with those of the United States and Canada (68–70); namely, Michaeli et al. (40) demonstrated that IPE was cost effective in primary and secondary prevention settings, but it offered more value in secondary prevention, with ICER of €18,133/QALY in patients without CVD vs. €14,485/QALY in patients with existing CVD over a 20-year time horizon. Importantly the number needed to treat was below six for nonfatal MI, nonfatal stroke, hospitalization for unstable angina, coronary revascularization, and CVD death in both primary and secondary prevention settings. In addition, the study showed that early lipid management with IPE starting at fifty-five years over twenty-five years could reduce ICER for primary prevention by nearly fifty percent to €9,381/QALY (40).

The Israeli health care perspective echoed that of the United States, Canada, and Germany, showing that prioritizing IPE therapy for patients with established CVD over primary prevention avoids fourfold higher rates of major adverse CV events for resources spent (66).

The cost effectiveness of IPE from an Australian health care perspective was dependent on the drug cost utilized and model inputs. Gao et al. (72) found that IPE for secondary prevention was not cost effective at a willingness-to-pay threshold of AUD50,000. However, Ademi et al. (71) found that it was cost effective for secondary prevention alone and prevented 270.8 MIs, strokes, or both; 295.8 coronary revascularizations; and 57.8 deaths overall vs. a statin alone. Differences in these study results were attributed to variation in annual cost of IPE (AUD3,768 vs. 1,637) and discount rate (3% vs. 5%) (71). Discrete patient-level data from REDUCE-IT were not used for either study, as was done in the primary cost-effectiveness analysis from REDUCE-IT (69).

## 4. Discussion

Studies of IPE eligibility across geographies demonstrate that a considerable portion of patients with residual persistent CV risk could benefit from treatment with IPE in primary and secondary prevention settings, ranging from 2.8% to 45.3% (47, 48). Variation in IPE eligibility largely depended on whether studies used highly selected patient populations (eg, history of CABG) vs. broader populations (eg, general adult population); which and how many of the REDUCE-IT, regulatory label, or guideline criteria were used; and whether studies assessed IPE eligibility in primary, secondary, or both prevention settings. Importantly, some of the most common reasons for IPE ineligibility were that patients did not meet TG and LDL-C criteria (41, 47, 48), suggesting that existing lipid-lowering therapies such as statins remain underused (59).

Studies assessing cost effectiveness worldwide found that IPE was generally most cost effective for secondary prevention. Several studies found that IPE is cost effective in both settings. Variations in the cost effectiveness of IPE across studies are largely attributed to differences in regional drug pricing and modeling assumptions. A majority of studies used a willingness-to-pay threshold of $50,000, whereas several used $100,000. Identifying which populations may benefit more from IPE and in whom IPE would be a more cost-effective treatment are important considerations given health care budget constraints (39, 40, 68–72).

Bolstered by the regulatory approval of IPE and widespread adoption of IPE recommendations into international medical society scientific statements and guidelines (31, 33–38), global findings from IPE eligibility and cost-effectiveness studies further underscore that IPE is a feasible add-on treatment to statins, especially in the secondary prevention setting. Importantly, the benefits of IPE cannot be generalized to other formulations of EPA. This is important because IPE is not available in some countries.
